# Natural Products as Outstanding Alternatives in Diabetes Mellitus: A Patent Review

**DOI:** 10.3390/pharmaceutics15010085

**Published:** 2022-12-27

**Authors:** Ingrid Andrea Rodríguez, Mairim Serafini, Izabel Almeida Alves, Karen Luise Lang, Fátima Regina Mena Barreto Silva, Diana Marcela Aragón

**Affiliations:** 1 Departamento de Farmacia, Facultad de Ciencias, Universidad Nacional de Colombia, Bogotá 110321, D.C., Colombia; 2 Departamento de Farmácia, Universidade Federal de Sergipe, Sao Cristovao 49100-000, SE, Brazil; 3 Department of Medicines, Faculty of Pharmacy, Universidade Federal da Bahia, Salvador 40170-115, BA, Brazil; 4 Departamento de Farmácia, Campus Governador Valadares, Universidade Federal de Juiz de Fora, Governador Valadares, Juiz de Fora 36038-330, MG, Brazil; 5 Departamento de Bioquímica—Centro de Ciências Biológicas, Universidade Federal de Santa Catarina, Rua João Pio Duarte Silva, Florianópolis 88037-000, SC, Brazil

**Keywords:** diabetes mellitus, natural products, flavonoids, polyphenols, patent

## Abstract

Diabetes mellitus (DM) is a metabolic syndrome that can be considered a growing health problem in the world. High blood glucose levels are one of the most notable clinical signs. Currently, new therapeutic alternatives have been tackled from clinicians’ and scientists’ points of view. Natural products are considered a promising source, due to the huge diversity of metabolites with pharmaceutical applications. Therefore, this review aimed to uncover the latest advances in this field as a potential alternative to the current therapeutic strategies for the treatment of DM. This purpose is achieved after a patent review, using the Espacenet database of the European Patent Office (EPO) (2016–2022). Final screening allowed us to investigate 19 patents, their components, and several technology strategies in DM. Plants, seaweeds, fungi, and minerals were used as raw materials in the patents. Additionally, metabolites such as tannins, organic acids, polyphenols, terpenes, and flavonoids were found to be related to the potential activity in DM. Moreover, the cellular transportation of active ingredients and solid forms with special drug delivery profiles is also considered a pharmaceutical technology strategy that can improve their safety and efficacy. From this perspective, natural products can be a promissory source to obtain new drugs for DM therapy.

## 1. Introduction

Diabetes mellitus is a metabolic disbalance among carbohydrates, lipids, and proteins that are associated with hyperglycemia, cardiovascular events, kidney, and ocular perturbances, peripheral neuropathy, ulcers, and even lower limb amputations [1,2,3]. A defect in insulin secretion and/or action is identified as one of the pathogeny causes [4]. Type 2 diabetes is the most reported form [5]. It occurs when the insulin levels decrease progressively, and the insulin resistance increases. Furthermore, type 1 diabetes is defined as a chronic condition in which the pancreas produces low levels of insulin or almost none [6].

The International Diabetes Federation reports estimate that about 537 million people live with DM worldwide. The countries with the highest incidence levels are by China, India, the United States, Pakistan, and Brazil [7]. In addition, low- and middle-income countries have the largest number of DM cases, and 1.5 million deaths are directly caused by diabetes each year. In recent decades, new case numbers and prevalence indicators in DM have been increasing. This has triggered sanitary alerts in international health systems, stimulating the research and development of new therapeutic strategies.

Currently, there are different treatments for DM type II, including changes in lifestyle, the use of oral hypoglycemic drugs or biotechnology products, gene therapy, pancreatic transplants, and precision medicine, to personalize diabetes prevention and management [8,9,10,11,12]. However, it has recently been shown that natural products and isolated substances play an important role in DM [13,14,15,16,17].

**Figure 1:**
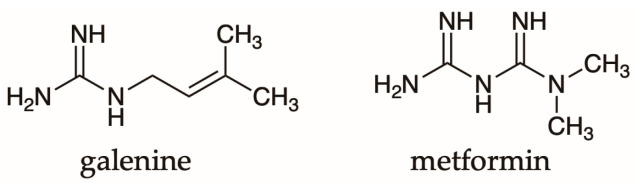
Chemical structure of galegine and metformin.

*Galega officinalis* L. (Fabaceae) was one of the first medicinal plants associated with antidiabetic activity prescribed in the Middle Ages [18]. Galegine metabolite was isolated from this plant, which is also known as galega or goat’s rue. This substance corresponds to a guanidine derivative with high toxicity levels [19]. However, this important fact helped to establish a precursor prototype to develop metformin the first hypoglycemic drug of the biguanide type. Their structures are shown in Figure 1.

Pycnogenol (acquose extract derived from *Pinus pinaster* (Pinaceae)), acarbose, miglitol, and voglibose (α-glucosidase inhibitors isolated from microorganisms) are other examples of natural products applied in DM treatment [20,21]. Ethnopharmacology criteria represent a valuable tool that allows advancing systematic analysis of natural products as innovative drugs in the industrial application field. A systematic patent review was carried out in the 2016–2022 period (Table 1) in which research with therapeutic DM potential based on natural products was selected. *Curcuma longa, Trigonella foenum-graecum,* and *Panax ginseng* were the main assessed as well as seaweed, fungi, and several minerals’ derivatives. Among the therapeutic targets, the inhibition of α-amylase, effects on glucose uptake, effects on glucose transporters, improvement in insulin secretion, the proliferation of pancreatic β-cells, and inhibition of proteins such as tyrosine phosphatase were found [15,22,23,24].

Furthermore, organic acids, polyphenols, saponins, terpenes, and flavonoids were identified as the responsible metabolites for the potential activity. Mainly, improved insulin signaling, effects on lipids, reduction in fat accumulation, inhibition of α-glycosidase activity, an influence on glucose metabolism, antioxidant activity, and influence on the intestinal microbiota were the effects derived from their use. Likewise, some pharmacotechnical strategies such as solid forms (tablets and granules), liquid forms (syrups and solutions), heterodisperse systems (suspensions), and transmucosal adhesive forms were also discussed. Finally, safety assessments and toxicity tests (in vitro and in vivo), and clinical efficacy reports were included.

## 2. Materials and Methods

Espacenet developed by the European patent office (EPO) was chosen as a database repository. The keyword diabetes in the title or abstract and the code a61k36/00 were chosen as the inclusion criteria. According to the International Patent Classification (IPC), the code a61k36/00 is associated with medicinal preparations of the indeterminate constitution, which contain algae material, lichens, fungi or plants, or their derivatives, and traditional herbal medicines. The review process was carried out between February to April 2022.

By Espacenet, 183 patents satisfied the first criteria. Subsequently, 5 cases were duplicated, and they were rejected. The publication date was considered as the second criterion, and then the patents published before 2016 were also discarded (*n* = 144). Additionally, the titles and abstracts were read, and 3 of them were due to a lack of relation to DM.

**Figure 2:**
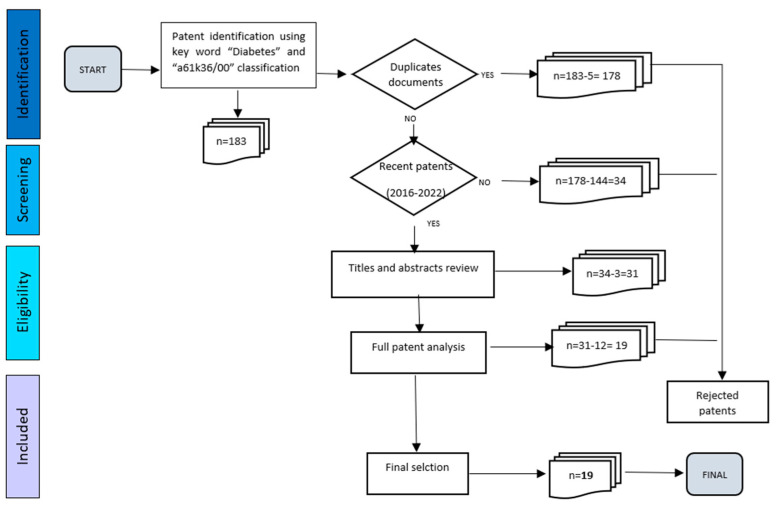
A systematic approach to the patent review with inclusion and exclusion criteria.

Then, 31 patents were fully analyzed, and 12 documents were out of the scope of this review. Finally, 19 documents were accomplished with all the criteria established. This allows the identification of natural products with potential applications in the pharmaceutical industry related to DM treatment. The screening scheme is represented in Figure 2.

## 3. Results

**Figure 3:**
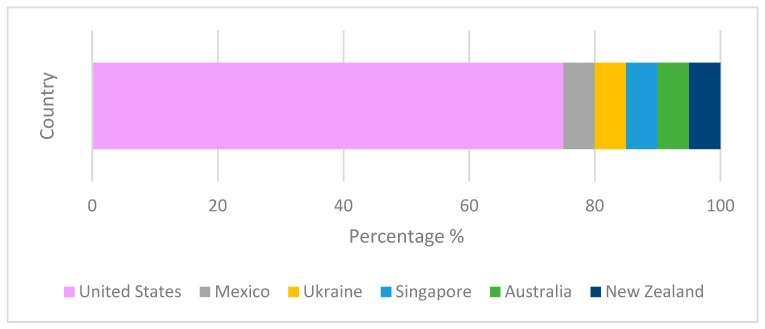
Patents for DM with A61K36/00 IPC classification that were published between 2016 and 2022. (100% represents the total number of 19 patents).

The United States, Mexico, Ukraine, Singapore, Australia, and New Zealand were the countries of origin of the reviewed patents. This can be observed in Figure 3.

**Figure 4:**
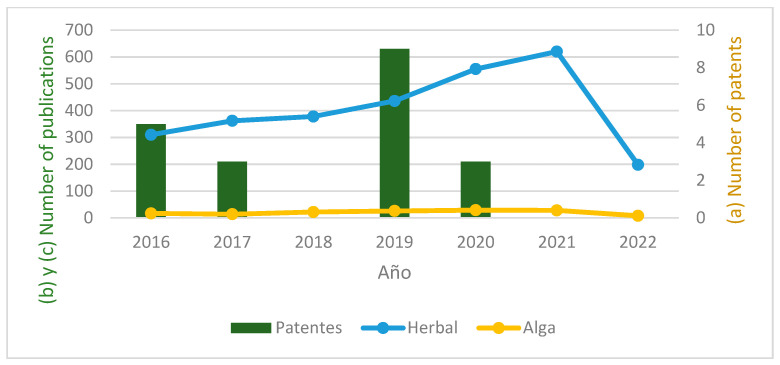
(a) Number of patents with IPC classification A61K36/00 published between period 01/2016 and 04/2022. (b) Number of articles published in PubMed between 01/2016 and 04/2022 associated with diabetes and herbal preparations. (c) Number of articles published in PubMed between 01/2016 and 04/2022 associated with diabetes and algae-based preparations.

The number of patents published and the scientific articles found in PubMed with keywords Diabetes (AND) herbal and Diabetes (AND) algae was also compared, as shown in Figure 4.

The worldwide impact of diabetes has increased in the latest years. Ongoing research work needs to be rigorous and constant to establish alternative therapeutic options. This context aims to analyze natural products with diverse and promissory mechanisms of action and their possible clinical applications.

This framework led to an in-depth investigation of the documents shown in Table 1. Plants, fungi, and seaweed extraction methods and some mineral compounds of traditional uses are compiled from patents in the field. Qualitative formula descriptions, pharmaceutical dosage form strategies derived from different extracts, and multi-component preparation (association of several natural derivatives materials) are also included.

## 4. Discussion

### 4.1. Main Hypoglycemic Mechanisms of Action

**Figure 5:**
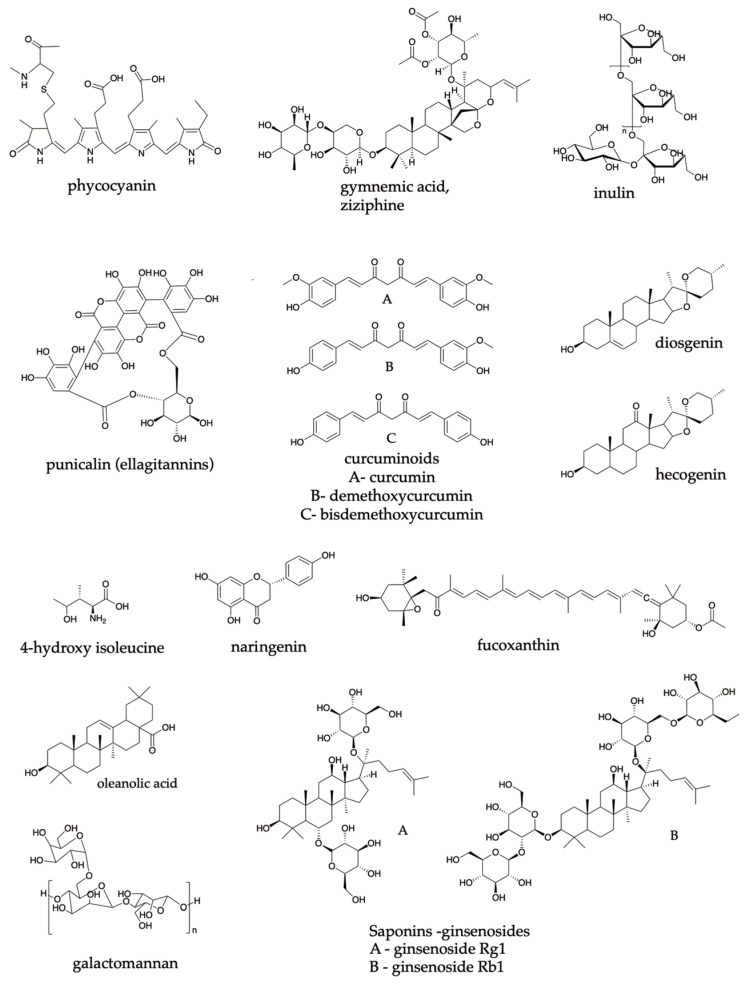
Principal metabolites reported by patents from natural products.

The multi-component systems are described in Table 1. Often, these formulations are made up of plants or a group of them. Therefore, some metabolites’ roles are highlighted in the same product. This is the case for SG11201908574T (A) patent, in which the ellagitannins (Figure 5) are the predominant substances with high maltase-inhibitory activity and energy expenditure (EE) increase by enhancing thermogenesis in brown adipose tissue [44,45]. Other activities derived from ellagitannins such as beneficial effects against insulin resistance, oxidative stress control in the polyol flow pathway, the activation of protein kinase C and the suppression of α-glucosidase, α-amylase, and lipase activities have been described [46,47,48].

Curcuminoids (Figure 5) are present in SG11201908574T (A) pharmaceutical preparation. Especially, bisdemethoxycurcumin [44] present in *Curcuma longa* has been related to the inhibitory activity of α-glucosidase derived from in vitro tests. Furthermore, some cellular and molecular effects have been attributed to these metabolites. For instance, a decrease in the sterol regulatory element-binding protein 1 (SREBP1c), and carbohydrate-responsive element-binding protein (ChREBP) and an increase in the carnitine palmitoyltransferase (CPT1) and the acyl-coenzyme A:cholesterol acyltransferase (ACAT) levels can allow the regulation of lipid metabolism [49]. An increase in PPAR-γ via AMPK activation and a decrease in lipid peroxidation effects are also associated with antioxidant activity with curcuminoids [50,51]. Finally, a decrease in monocyte chemoattractant protein-1 (MCP-1), IL-1β, TNF-α, IL-6, and COX-2, can be resulted in an anti-inflammatory effect [52,53,54]. Furthermore, an influence on the intestinal microbiota is also suggested, owing to a possible mediation in the metabolic syndrome process [55,56].

Meanwhile, different terpenes also have promising effects in DM treatment. Oleanolic acid is present in the species *Sambucus* spp. [57] has demonstrated an inhibitory activity on α-glucosidase, α-amylase, and protein tyrosine phosphatase 1B (PTP 1B) [58,59]. In this context, saponins or triterpenoids are also thought to be responsible for the beneficial effect in DM [60]. It was found that saponins such as kammogenin, manogenin, gentrogenin, and hecogenin (Figure 5) exhibited an α-glucosidase-inhibitory activity. They are included in the composition of the patent product MX2018004489 (A) [33]. A powerful hypoglycemic activity in an oral glucose tolerance assay in rats is also exhibited by these saponins [44].

SG11201908574T (A) patent refers to the presence of organic acids, which describe a potent antioxidant and hypoglycemic activity that can be evaluated in animal models of hyperglycemia [28,61]. The above also suggests stimulating effects on insulin secretion [44]. Likewise, it attenuates diabetic nephropathy through anti-inflammatory and antifibrotic effects, which are found in flavonoids such as the flavonone naringenin (Figure 5); this was identified in WO2019088958 (A2) and WO2019088958 (A3) patents [9,32]. These metabolites are important components of the human diet. and several studies have demonstrated a broad range of beneficial effects in DM, such as the balancing of glucose metabolism [14].

Some polysaccharides were described in the composition of the products in the US2016361341 (A1) and US9828442 (B2) patents. Decreased glucose absorption, increased serum insulin concentration, reduced postprandial hyperglycemia, and modulation of liver enzymes are the proposed mechanisms of action of these metabolites [40]. Inulin (Figure 5) is an example of these substances that can be applied to DM [9,44]. Additionally, it is known that oligosaccharides such as non-digestible carbohydrates, a subtype of this group of compounds, also play a role in modulating the intestinal bacterial population after dysbiosis [55,62].

**Figure 6:**
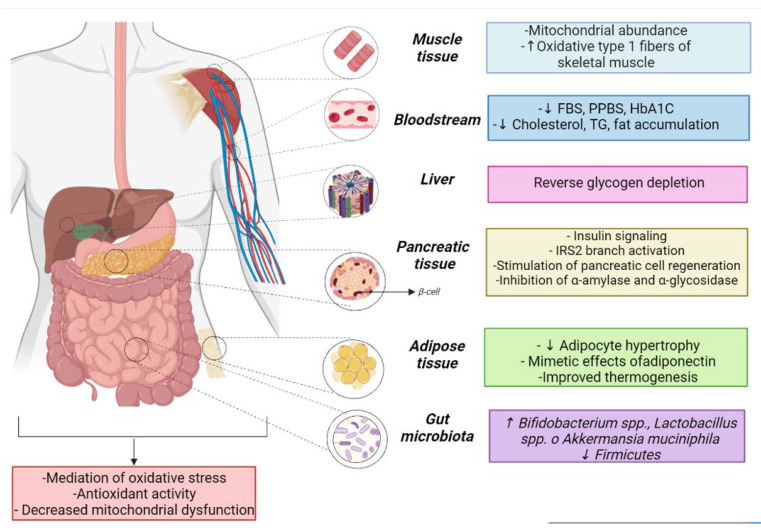
Mechanisms of action in DM related to natural products derived from the patent reviewed. (↑ increase ↓ decrease).

Different mechanisms of action derived from secondary metabolites are mentioned above in the reviewed patents. Nevertheless, an important number of patents do not mention the complete chemical composition. Only the species of plants, fungi, or seaweed and their parts such as roots, fruits, bark, leaves, heartwood, flowers, seeds, rhizomes, gum resin, or stems are detailed. The lack of information on the chemical identity in the preparation can be considered a limitation of the study. The poor description affects the monitoring of a particular pharmacological marker as being responsible for anti-DM effects. Even so, seven categories of various mechanisms of action that could contribute to the treatment of DM have been proposed (Figure 6) based on the authors’ annotations.

#### 4.1.1. Insulin Signaling

Anabolic processes comprise a series of harmonized steps that are oriented toward the construction of complex biomolecules. The procedure is carried out after the generation of covalent molecular bonds. As a consequence of these reactions, the reserve energy is available to be released according to the body’s demand [63].

Insulin is synthesized in the pancreas, a glandular organ located in the abdomen [64]. This hormone contributes to the anabolic process, which is mediated through glucose uptake in myofibrils and adipocytes [64]. DM cases are derived from the total or partial absence of insulin, which triggers a metabolic imbalance. As a solution, agents that promote or improve insulin signaling are considered a useful strategy in this field [65].

About 40% of the reviewed patent analyzed the improvement in insulin signaling as a benchmark derived from their preparations. For instance, an increase in insulin secretion and regeneration of pancreatic cells is mentioned in the US10576117 (B2) and US2019125816 (A1) patents [22]. This scenario is attributed to the use of *Gymnema sylvestre* extracts, whose major components are gymnemic acid, saponins, and gurmarin [22,66,67,68].

On the other hand, *Herbaceous Premna* and *Paiiurus spina-christi Mill* species are present in WO2020115767 (A1), WO2019088958 (A2), and WO2019088958 (A3) patents, in which the authors suggest an improvement insulin signaling via the AKT/AM PK pathway [25,69].

#### 4.1.2. Effects on Lipids

Lipidic perturbances are also characteristics of DM. As a result, a strong relationship between dyslipidemia, diabetes, and cardiovascular disease has been documented. It shows the metabolic interdependence between carbohydrate metabolism and lipids [70].

Higher levels of specific cholesterol subtypes and very low lipoproteins (VLDL), low-density lipoprotein (LDL), and triglycerides increase the likelihood of obstruction in vessels and capillaries, which promotes vascular changes. As result, this target can allow better lipid regulation and avoid complications resulting from DM [71].

In the WO2020115767 patent, a significant reduction in lipids was obtained. The decrease was induced by palmitate in a dose-dependent manner in HepG2 cells after *P. herbacea* administration. Then, it suggests the applicability of the herbal preparation to complications associated with hepatic insulin resistance in DM2 [25].

In the same way, the reduction in obesity, overweight, accumulation of visceral adipose fat and central obesity, adipocyte hypertrophy, intracytoplasmic accumulation of hepatic triglycerides, dyslipidemia, and LDL and the improvement in lipid degradation were also studied in the MX2018004489 (A), US10668122 (B2), and US2017252393 (A1) patents [33,37].

#### 4.1.3. Glucose Metabolism

Many natural products are useful in the control of DM due to their effects on glucose metabolism. The main ones are the action on degrading enzymes, glucose re-uptake, and agonist effects on specific receptors. During the digestion process, enzymes help break down macromolecules to make them available for absorption. One of them, α-glucosidase, helps in the cleaving processes of the starch molecules, allowing the absorption of released monosaccharides [72]. This fact explains the glucose bioavailability levels. The higher the α-glucosidase levels, the higher the probability of free glucose in blood circulation, which is critical in DM patients.

Digestive enzyme regulation can be considered a target of DM treatment. The possible delay of carbohydrate absorption in the intestine and the subsequent decrease in the postprandial insulin level [57] derived from enzyme control are highlighted by the US11007237 and (B2) US10640480 (B2) patents.

Additionally, the control of glucose levels may be mediated by peripheral glucose reuptake. Therefore, the glucose transporter (GLU), peroxisome proliferator-activated receptors (PPARs), and adipocytokines [72] have been described as targets of interest.

For instance, adiponectin (a type of adipocytokine) represents a protein involved in insulin sensitivity, glucose uptake, and lipid metabolism. Chronic inflammatory processes can be expressed in DM due to low serum levels of this substance [73]. In addition, osmotin is described as a structural homolog of the β-barrel domain of adiponectin in WO2019186579 (A1). This enzyme can bind and activate the adiponectin receptor (ADIPOR1), which led carrying out kinase phosphorylation through AMPK activation (3′ 5′- adenosine-monophosphate-kinase) [74]. The previous process takes place on muscle cells and has a beneficial effect on DM treatment.

Nonetheless, natural products can strengthen the pancreatic tissue and promote insulin secretion and decrease the intestinal absorption of glucose [9].

*Trigonella foenum-graecum* species present in two patents contain metabolites such as diosgenin, galactomannan, and 4-hydroxy-isoleucine (Figure 5) that have been associated with lowering blood glucose levels and insulin sensitivity [75].

Ginsenosides (Figure 5) from *Panax ginseng* have preclinical and clinical reports reflecting the improvement in glucose metabolism and renal function [22,75]. Moreover, the European Medicines Agency (EMA) established its inhibitory effect on the plasma glucose-lowering action of Rh2 by opioid µ-receptor blockers [76].

#### 4.1.4. Influence on the Intestinal Microbiota

The relationship between the gut microbiota, the onset of insulin resistance, and diabetes has been investigated for years. Intestinal permeability, endotoxemia, interaction with bile acids, and changes in the proportion of adipose tissue are possible mechanisms of interconnection [77]. An increase in the abundance of Firmicutes phylum bacteria and a reduction in Bacteroidetes phylum bacteria have been reported in people with obesity and DM [78]. However, natural products can ameliorate the control and balance of microorganisms’ growth and strengthen intestinal health.

Some polyphenols of natural origin have probiotic activity and can influence the intestinal microbiota, being benefic for obesity and metabolism. That is the case of curcumin (Figure 5), a representative metabolite of *Curcuma longa.* Intestinal barrier function, reduced circulating LPS levels, and improves glucose tolerance in LDLR 1 were found after the oral administration of curcumin in mice [55].

A possible reduction in the imbalance of the Firmicutes/Bacteroidetes ratio in the gastrointestinal tract was researched in WO2019205943 (A1), which is derived from various plants, fungi, or their association [29,79]. Likewise, several beneficial effects such as a decrease in Firmicutes and an increase in Bacteroidetes, *Lactobacillus* spp., *Bifidobacterium* spp., and *Akkermansia muciniphila* are also discussed in MX2018004489 (A). The microbiota perturbances are related to mucin degradation, which represents a protective glycoprotein in the intestinal mucus layer and has been associated with better insulin sensitivity and decreased fat gain [33,36].

#### 4.1.5. Oxidative Stress

High levels of glucose in DM may be associated with the presence of reactive oxygen species (ROS) due to the degradation of LDL lipids [75]. The presence of ROS triggers chemical reactions binding to lipid and protein membranes. The disruption of physiological conditions exacerbates oxidative damage and modifies the cellular environment [72]. The conditions mentioned result in an insulin resistance scenario. The antioxidant activity is discussed in several patents. Limiting the propagation of radicals and interrupting membrane damage are characteristics of these processes.

The improvement in pancreatic β cell function and regulation of insulin tolerance has been explained as an antioxidant and antidiabetic effect from curcumin in the patent review [75]. Meanwhile, polyphenols in *Vitis vinifera* (patent WO2019186579 (A1)) were associated with antioxidant capacity and insulin resistance decrease [30,75]. *Moringa oleifera* and *Zingiber officinale* also contribute to the mitigation of oxidative stress in animal models [22]. In addition, cyanobacterial-derived metabolites, such as phycocyanin (Figure 5), act against oxidative damage and contribute to preventing chronic inflammation [31].

#### 4.1.6. Other Mechanisms

Thermogenic effects are also found as a pharmacological strategy favorable in DM cases. It is linked to the body’s ability to generate heat after metabolic reactions. Free fatty acid concentration and insulin sensitivity are associated with an improvement in metabolic parameters through the activation of brown fat [80]. Thermogenic effect is mentioned on MX2018004489 (A), in which the inhibition of phosphodiesterase activity is carried out [33]. In addition, stimulating PGC-1Q and UCP1 and activating AMPK [41] mechanisms are shown in US10028930 (B2) and US2016228400 (A1) as a result of thermogenic effects.

Other documents, such as US10576117 (B2) and US2019125816 (A1), describe the reversing processes of glycogen depletion and proteins that are generally observed in the tissues of diabetic subjects [36].

### 4.2. Perspectives for Pharmaceutical Dosage Forms

Conventional pharmacotherapeutic schemes in DM include the daily administration and multiple doses of several drugs [81]. These facts can transform into disadvantages such as adverse effects and difficulties in adherence to therapy [75]. Consequently, providing adequate and effective clinical strategies is a constant challenge in pharmaceutical research.

In this sense, the application of controlled release systems is thought to be a promising approach to reducing the number of drugs in DM treatment [82]. This technology requires the critical choice of excipients, which will support the drug release according to the physiological conditions. As result, dose–response curves and retention times of the drugs can be adjusted to contribute to the pharmacokinetic parameters and reduce drug ingestion.

For instance, a protective bilayer dosage form is designed in the S10028930 (B2) and A US2016228400 (A1) patents. In this case, thermogenic substances such as fucoxanthin and caffeine are released with an hour of difference, which can increase energy expenditure and lipid oxidation [83]. It even suggests a dual-acting anti-diabetic and anti-hypertensive derived from this type of metabolite [84]. Another method is related to the administration of active salt forms that exert their action according to the pH of the medium. Therefore, drugs are often chemically made into their salt forms to enhance how the drug dissolves, boost its absorption into the bloodstream, and increase its effectiveness [85]. Finally, prodrugs are also included in the patent review. They can undergo chemical pH-dependent transformations, which allow extended time activation according to the medium.

A transdermal patch with a prodrug system was discussed in the US10028930 (B2) and A US2016228400 (A1) patents. The prodrug-to-drug conversion was performed due to an enzyme or chemical reagent included in the matrix [41]. Additionally, tablets or pills containing the extract of *Calophyllum inophyllum* were also proposed by NZ630125 (A). The invention performs a modified coating that prolongs the action of metabolites in DM treatment.

Otherwise, several inventions do not detail the delivery systems in which extracts are incorporated. For example, US10799547 (B2) and US2016067294 (A1) patent presents a wide range of forms, including granules, powders, syrup, solutions, suspension, tablets, injectables, poultices, and capsules to transport *Suaeda japonica* extract. These dosage forms realize different release forms such as rapid, continuous, or delayed [42].

In WO2019186579 (A1) patent, structural homology between osmotin and adiponectin domain, provides a possibility of a controlled release system through different pharmaceutical forms, such as tablets, granules, syrups, and films for transmucosal administration [86].

On the other hand, mucosal films have been tackled in patents. As part of improving the dissolution drug in the oral cavity, a transmucosal film of metformin and linagliptin was carried out. The omission of first-pass metabolism, a shorter time, and a faster-acting response compared to conventional pharmaceutical forms were the main advantages of this technology [87,88].

Other findings in the patent review are oriented to the optimization of extraction processes to maximize yields and achieve a better loading of the pharmaceutical vehicle. An extraction solvent based on an emulsifier, cyclodextrin, acid, water, and/or an organic solvent was related to *Gnetum gnemon* extract by the US10640480 (B2) and US2019031635 (A1) patents. This solution boosts the gnetin C glycoside content due to the solvolysis reaction [34].

### 4.3. Safety Test Assessments

Alternative therapies for DM from natural origins must follow the non-maleficence principle, in which the new substance administration does not represent a health risk. Safety in this field is based on the correct tracking of metabolites, their quantification, and the correct doses administrated. The current open science idea increases the safety background to divulge key data from diverse natural products. This fact mitigates the premature use of raw materials and unreliable data in natural products [72]. In this sense, acute and chronic toxicity studies are considered valuable tools to analyze the possible adverse effects derived from natural products.

The results obtained in these tests can guide the next steps in the research perspective. For instance, particular findings were obtained in the in vivo acute toxicity test performed in SG11201908574T (A). Inventors mentioned behavioral changes from 30 min until the 15th day after oral administration, characterized by tremors, sedation, convulsions, catatonia, state alertness, muscle spasms, cyanosis, writhing, irritability, and diarrhea.

On the other hand, lethal doses (LD) are also a parameter studied in the safety stage. For instance, greater LD values of 5000 mg/kg in rats were demonstrated in a safety assessment (short and long-term) in the US10576117 (B2) product.

Nevertheless, this information must be tackled carefully. Curcuminoids (present in several patents) have been identified as hepatotoxic in rodents [89,90]. The toxicity was linked to the metabolic pathways, especially 3,4-epoxidation predominant in rats. However, in humans, the metabolism occurs through the 7-hydroxylation pathway, which indicates that the hepatotoxic metabolites are not formed [72,91,92]. In contrast, these human metabolites are related to minor symptoms, such as itching, constipation, vertigo, and diarrhea [52].

Other safety warnings in natural products are focused on terpenes. Some terpenes have high molecular weights, and their permeability properties can be limited in physiological conditions. Although some tests suggest their accumulation in the liver, which can be dangerous, other researchers do not indicate adverse effects after acute and chronic ingestion [57]. Furthermore, a co-administration therapeutic regimen can also be reviewed. The concomitant use of traditional drugs and natural products may result in complications. An example can be the water-soluble fraction of okra (*Abelmoschus officinalis*) fruits, which modified the metformin absorption, reducing its serum concentration [72].

### 4.4. Pharmacological Assessment

To confirm the benefits of DM therapy, the preparation must be challenged to guarantee its effectiveness. This ensures that the mentioned therapeutic targets are achieved.

Despite the number of biochemical or cellular assays to test the target activity, the (AU2018278958 (A1) and AU2018278958 (B2) patents describe an alternative assay. The applicants have created a system in which a cell-specific protein-based target can identify activators of the IRS2 branch in the insulin-mediated signal transduction cascade. To support the procedure, control and test cells were derived from the 32D myeloid progenitor cell line [35,93].

However, the in vivo models are still predominant. To model DM, the use of streptozotocin (STZ) and alloxan (ALX) were mentioned by several patents and literature reviews [94,95]. These substances easily accumulate in pancreatic β-cells through the glucose transporter GLUT2. The STZ model is characterized by the presence of methylnitrosourea. This metabolite modifies biological macromolecules, fragments DNA, and destroys β-cells. Meanwhile, the ALX model has an oxidative stress capacity mediated by dialuric acid. In this case, the metabolite activates ROS cascades, impacting β-cell dysfunction [96].

Other findings in in vivo models indicate the use of OLETF (Otsuka Long-Evans Tokushima Fatty) mice, which are mentioned in the US10799547 (B2) and US2016067294 (A1) documents. The DM model is performed with a hypercaloric diet. When the DM model is stabilized, the extract is administered. Then, the blood and plasma samples are tracked to determine their content in the body.

Once the in vitro and in vivo stages are optimized, the next step is to carry out clinical trial assessments [52]. Only 15% of patients reported clinical studies annotations.

The influence of Ayurvedic medicine was observed in the clinical tests of DM in the patent review. The product administration was carried out in a personalized way. According to the Ayurvedic standard, dose adjustment and regimen timelines of administration led to the clinical criteria for practitioners to use the inventions [36].

To illustrate this practice, the US10028930 (B2) and US2019125816 (A1) patent inventors performed a prospective, open-label, non-randomized, phase III clinical trial. The procedure took place in the Muniyal Ayurvedic Hospital and Research Center in Manipal, India, and the patients were individuals from 30 to 60 years old [36]. London protocol elements such as inclusion and exclusion criteria or voluntary withdrawal were also documented. Despite the clinical trial details not being shown, the aim of the practice was clear, namely to verify the effectiveness [41].

Another example of clinical trials in this review refers to the use of *Trigonella foenum-graecum* in DM. Nevertheless, a lack of harmonization in the administration schedule was identified. This framework suggests an improvement opportunity for variable control in further assessment to reach a convergence protocol [9].

Although clinical assessments must be carried out within the transparency standards (under compliance agreements), the status of their execution must be critically monitored. Government entities such as the National Institute of Health (NIH) record the execution and status of these studies. The information is saved in an online database (https://clinicaltrials.gov/) and available to the general public. There are about 50 active trials related to DM and herbal components, predominantly located in China, India, and the United States [97].

### 4.5. Other Natural Resources with Anti-DM Potential

To achieve a therapeutic effect, WO2019205943 (A1), US10028930 (B2), US2016228400 (A1), US2016361341 (A1), and US9828442 (B2) describe the use of extracts and metabolites from algae, fungi, and metals or their derivatives.

Fucoxanthin is a carotenoid extracted from brown algae. Their effects in DM are observed in US10028930 (B2) and A US2016228400 (A1) [41]. Previous reports of this algae-derived metabolite have highlighted its role in inducing UCP1 in the white adipose tissue mitochondria. Fatty acid oxidation, heat production, insulin resistance improvements, and decrease blood glucose levels are the main effects of their use [98].

Additionally, the *spirulina cyanobacterium* metabolite phycocyanin has powerful antioxidant, anti-inflammatory, adipogenesis- reducing and thermogenic-activating properties that can be considered in this field [99,100]. Thus, their presence increases the body’s ability to prevent the development of chronic inflammation, which is frequent in DM patients [31].

Similarly, polysaccharides isolated from *Hirsutella sinensis* (fungi) improved insulin sensitivity in rodents in in vivo studies [40]. Increased expression of protein markers of thermogenesis, intestinal integrity improvements, anti-inflammatory effects, and lipid metabolism regulation were found for this substance [101,102].

Regarding Ayurvedic medicine influence, the shilajit (asphaltum black) a complex derived from the decomposition of plant and mineral materials was deepened by US 10967025 (B2) and US2019209634 (A1) [103]. The complex contains more than 85 minerals, as well as humic acid, dibenzo-α-pyrones, and fulvic acid. Antioxidant and cytoprotective properties are associated with it, which can be exploited in the prevention of DM disease [31].

In the same way, the use of minerals such as mica, pyrite, tin, lead, zinc, coral, iron, and copper and bhasmas or calcined preparations such as Swarna Makshika Bhasma, Abhraka Bhasma, Loha Bhasma, Vanga Bhasma, Yashada Bhasma and Pravala Bhasma derived from Ayurvedic medicine were reported in US10576117 (B2); US2019125816 (A1) Nevertheless, the literature available in online databases is focused on the synthesis and physicochemical characterization of these Herbo-Mineral preparations [36,104,105,106,107]. Therefore, further research on the subject is required.

## 5. Conclusions

This review shows different methods of preparation and extraction of natural products from plants, algae derivatives, fungi, and some minerals. The most reported and studied secondary metabolites in patents are tannins, organic acids, polyphenols, saponins, terpenes, and flavonoids. Among the mechanisms of action, the effects on the circulatory system, intestines, pancreas, adipose tissue, and muscles have been identified.

Additionally, some pharmacotechnical strategies such as solid forms with a modified release, bilayer systems, and transmucosal films are reported. Finally, some key safety and efficacy points are discussed, which provide a critical analysis of these substances in the current context.

The above demonstrates the importance of research using medicinal plants in the design and development of new drugs for the treatment of DM.

## Figures and Tables

**Table 1:** Pharmaceutical product patents with natural origin published by EPO from 2016 to 2022.

Year	Country	Registration Number	Species	Part of the Plant	Secondary Metabolites	Mechanism of Action	Formulation	Safety Assessments	Efficacy Tests	Ref
2020	WO	**WO2020115767 (A1)**	Premna herbacea	Leaves	isoverbascoside	Improved insulin signaling via the AKT/AM PK pathway	ND	A single dose of 2000 mg/kg of *P. herbacea* methanol extract was reported as safe after acute toxicity evaluation in Swiss ICR male mice.	In vitro: [a] Uptake of glucose in L6 cells [b] Total accumulation of lipids in HepG2 cell lines by staining with oil red 0 In vivo: ND Clinical studies: ND	[25]
2020	US	**US11007237 (B2); US2020147160 (A1)**	*Pinus* spp.	Resin	ND	α-glucosidase inhibition	Syrup, tablets, capsules, granules, and powders.	ND	In vitro: [a] Suppression of α-glucosidase activity [b] Ability to uptake glucose Clinical studies: ND	[26]
2020	WO	**WO2020012299 (A1)**	*Curcuma longa, Phylanthus emblica*	ND	ND	Insulin sensitization and antihyperglycemic	ND	Efficacy studies were carried out with non-cytotoxic doses after evaluation of cytotoxicity and PPAR γ expression in fibroblasts using the MTT assay.	In vitro: [a] Evaluation of PPAR γ expression in fibroblasts cell culture. In vivo: Evaluation of streptozotocin-induced insulin resistance in rats. Clinical studies: ND	[27]
2019	SG	**SG11201908574T (A)**	*Curcuma longa, Emblica officinalis, Vernonia anthelmintica, Tinospora cordifolia, Trigonella foenum-graecum, Ixora coccinea, Syzygium cumini*	ND	Tannins, organic acids, curcuminoids	ND	Powders, pastes, granules, capsules, tablets, emulsions, suspensions, syrup, elixir, oral drops, and nutraceuticals	Acute toxicity test in male and female Swiss ICR mice, at doses. It was reported as safe.	In vitro: [a] Evaluation of glucose uptake potential in L6 cells [rat skeletal muscle cells] In vivo: ND Clinical studies: ND	[28]
2019	WO	**WO2019205943 (A1)**	*Polygonatum* spp., *Poria cocos, Lycium barbarum, Pueraria Panax ginseng, Shiitake, cordyceps, Ganoderma lucidum, Hericium erinaceus,* *Tremella* *Dendrobium officinale*	ND	Flavonoids and oligosaccharides (inulin, fructooligosaccharides, oligosaccharides)	Reduction in the proportion of Firmicutes/Bacteroides in the gastrointestinal tract.	ND	The description of the methodology was not detailed, but it was concluded that the preparation is safe.	In vitro: AND In vivo: Oral glucose tolerance test in different groups of mice. Clinical studies: ND	[29]
2019	WO	**WO2019186579 (A1)**	*Trigonella foenum-graecum, Solanum lycopersicum, Dudhi bhopla, Lagenaria siceraria, Vitis vinifera, Medicago sativa*	Roots, stems, leaves, buds, flowers, fruits, and seeds.	Osmotin	Adiponectin agonists/mimetics. Activation of the AMPK pathway	Tablets, syrup, granules, and film for transmucosal administration.	ND	In vitro: ND In vivo: ND Clinical studies: ND	[30]
2019	US	**US10967025 (B2) US2019209634 (A1)**	*Moringa Oleifera, Origanum vulgare, Shilajit, Blue-Green Algea, Phyllanthus emblica, Piper nigrum, Salvia rosmarinus, Punica granatum, Trigonella foenum-graecum, Curcuma longa.*	Leaves, fruits, and seeds.	Polyphenols, carvacrol, phycocyanin	Antioxidant and anti-inflammatory—via COX-1 inhibition	Capsules	ND	In vitro: [a] ROS absorption capacity [b] DPPH radical scavenging activity [c] Oxidation of LDL in a cell-free system [d] Insulin release in islets of rats [e] Effect on cholesterol absorption in Caco-2 cells [f] Effect on COX-1 Enzyme activity. In vivo: ND Clinical studies: ND	[31]
2019	WO	**WO2019088958 (A2); WO2019088958 (A3)**	*Paiiurus spina-christi*	Fruits	Naringenin	Effect on ALT and AST regulation enzyme levels, antioxidant activity, increased insulin secretion, regulates blood glucose, and increased magnesium absorption	ND	ND.	In vitro: ND In vivo: Rats with streptozotocin-induced diabetes Clinical studies: ND	[32]
2019	MX	**MX2018004489 (A)**	*Agavaceae* spp.	Leaves, rhizomes, and mead	Steroidal saponins and sapogenins (kammogenin, manogenin, gentrogenin, and hecogenin).	Lipid and glucose metabolism improvements, energy expenditure, gut microbiota health, muscle oxidative capacity, and thermogenesis, stimulating PGC-1Q and UCP1, and activating AMPK	Pills, capsules.	ND	In vitro: ND In vivo: ND Clinical studies: ND	[33]
2019	US	**US10640480 (B2); US2019031635 (A1)**	*Gnetum gnemon*	Fruits	Gnetin C	Antioxidant, antibacterial, inhibition of lipase enzymes, α-glucosidase, and α-amylase.	Powders, granules, tablets, capsules.	ND	In vitro: ND In vivo: ND Clinical studies: ND	[34]
2019	AU	**AU2018278958 (A1); AU2018278958 (B2)**	*Cichorium endivia, Latifolium; Lactuca sativa, Longifolia; Lactuca sativa*. Plantas das famílias *Asteraceae* spp., *Lamiaceae* spp., *Brassicaceae* spp., *Amaranthaceae* spp.	Fruits, leaves, stems, and roots	ND	IRS2 branch activation of the insulin-mediated signal transduction cascade.	N.D.	ND	In vitro: ND In vivo: ND Clinical studies: ND	[35]
2019	US	**US10576117 (B2); US2019125816 (A1)**	*Salacia chinensis, Gymnema sylvestre, Emblica officinalis, Eugenia jambolana, Curcuma longa, Commiphora mukul, Tinospora cordifolia. Ithania somnifera, Terminalia chebula, Andrographis paniculata, Boerhavia diffusa, Azhadirachta indica, Aristolochia indica, Aegle marmelos, Cyperus rotundus, Hemedesmus indicus, Trichosanthes dioica, Santalum alba, Terminalia arjuna, Woodfordia fruiticosa, Glycerrhiza glabra, Mucuna pruriens, Myrica nagi, Plumbago rosea, Inula racemosa, Zingiber officinalis, Piper longum y Piper nigrum*	Roots, fruits, bark, leaves, heartwood, flowers, seeds, rhizomes, gum resin, and stems.	ND	Lipids/cholesterol reduction, tissue phosphatases, and tissue transaminases reduction, reversal of glycogen depletion, pancreatic islets cell regeneration, hypoglycemic, hypolipidemic, cytoprotective, and immunomodulatory action. FBS, PPBS, HbA1C reduction	Tablets, granules, capsules, solution, emulsion, suspension	According to OECD guidelines in rodent tests 423, 407, 408, and 452. It was reported as safe.	In vitro: ND In vivo: Model of diabetes in Wistar rats diabetic by alloxan. Clinical studies: A prospective, open-label, non-randomized, phase III clinical trial conducted in outpatients at Muniyal Ayurvedic Hospital and Research Centre, Manipal, India.	[36]
2017	US	**US10668122 (B2); US2017252393 (A1)**	*Toona sinensis*	leaves	Monoterpenes, diterpenes, triterpenes, sesquiterpenes, saturated and unsaturated fatty acids	Glucose absorption improvements, lipid degradation, inhibition of large lipid clusters, and inhibition of metabolic syndrome	ND	ND	In vitro: 24 h glucose consumption of 3T3-L1 adipocyte culture medium. 2- Adipogenesis blocked by TS-SCF in adipocytes 3- Antidiabetic effect of high to medium/high polar components of TSL, except TSL-SCF In vivo: ND Clinical studies: ND	[37]
2017	US	**US10111923 (B2); US2017173103 (A1)**	*Cyclocarya paliurus*, *Puerariae lobatae Radix, Polygonati odorati Rhizoma. L*	ND	ND	ND	Pills, capsules, granules, powders, or tea formulation	ND	In vitro: ND In vivo: Rats with streptozotocin-induced diabetes Clinical studies: ND	[38]
2017	NZ	**NZ630125 (A)**	*Calophyllum inophyllum*	Cortex	ND	Inhibition of DGAT-1 and SCD-1 enzyme activity	Tablets, coated tablets, capsules, powders, granules, elixir, and syrup.	ND	In vitro: 1- Assay of DGAT-1. 2- Inhibition of hDGAT-1 3- SCD-1 Assay 4- Triglyceride synthesis assay in HepG2 cells In vivo: Streptozotocin-induced diabetic rats fed a high-fat diet. Clinical studies: ND	[39]
2016	US	**US2016361341 (A1); US9828442 (B2)**	*Hirsutella sinensis*	ND	Polysaccharides	Hyperglycemia reduction and improvement of insulin sensitivity in humans and animals.	ND	ND	In vitro: ND In vivo: Streptozotocin-induced diabetic rats fed a high-fat diet. Clinical studies: ND	[40]
2016	US	**US10028930 (B2); US2016228400 (A1)**	*Ephedra sinica and* brown algae concentrate	ND	Fucoxanthin, caffeine, alkaloids such as ephedrine	Thermogenesis potentiator	Extended-release tablets, transdermal patches	ND	In vitro: ND In vivo: ND Clinical studies: ND	[41]
2016	US	**US10799547 (B2); US2016067294 (A1)**	*Suaeda japonica*	Leaves and seeds	ND	The cytoprotective activity of pancreatic β-cells, hypoglycemic action, insulin secretion increase, improvement of glucose tolerance, and increase of adiponectin content in the blood.	Pills, powders, granules, capsules.	ND	In vitro: ND In vivo: Overfeeding diabetic OLETF mice. ND clinical studies	[42]
2016	UA	**UA104161 (U)**	*Equiseti arvensis, Sambucus* spp., *Hypericum perforatum, Tiliae* spp., *Polygonum aviculare, Urticae* spp. *Inula helenium* e *Mirtilli* spp.	Flowers, leaves. Additional plant material: rhizomes and roots	Ascorbic acid, tannins, organic acids, flavonoids, hydroxycinnamic acids	Hypoglycemic action due to exposure to glucagon-like peptide 1 [GLP-1]. Increased insulin levels, improves carbohydrate and lipid metabolism.	ND	ND	In vitro: ND In vivo: Alloxan-diabetic female rats. Clinical studies: ND	[43]

Abbreviations: WO: World Intellectual Property Organization (WIPO). xUS: United States. SG: Singapore. MX: Mexico, AU: Australia. NZ: New Zealand.UA: Ukraine, AKT/AM PK: Protein kinase B or AKT / AMP-activated protein kinase. ICR: Cancer Research Institute. PPAR γ: Peroxisome proliferator-activated receptor. COX-1: Cyclooxygenase-1. ROS: reactive oxygen species. DPPH: 2,2-Diphenyl-1-picrylhydrazyl. LDL: low-density lipoproteins. ALT: alanine aminotransferase. AST: Aspartate aminotransferase. PGC-1Q: Peroxisome proliferator-activated gamma recipe coactivator. UCP1: Uncoupling protein 1 or thermogenin. IRS2: insulin receptor substrate 2. FBS: Fasting blood sugar. PPBS: Postprandial blood sugar test. HbA1C: glycosylated hemoglobin. TS-SCF: T. sinensis- supercritical carbon dioxide fluid. TSL-SCF: Hojas de T. sinensis- supercritical carbon dioxide fluid. DGAT-1: Diacylglycerol O-acyltransferase 1. Human hDGAT-1: Diacylglycerol Acyltransferase -1. SCD-1: stearoyl-CoA desaturates.

## Data Availability

Not applicable.

## References

[1] (2009). Association Diabetes American Diagnosis and classification of diabetes mellitus. Diabetes Care.

[2] Maida C.D., Daidone M., Pacinella G., Norrito R.L., Pinto A., Tuttolomondo A. (2022). Diabetes and Ischemic Stroke: An Old and New Relationship an Overview of the Close Interaction between These Diseases. Int. J. Mol. Sci..

[3] WHO Diagnosis and Management of Type 2 Diabetes (HEARTS-D). https://www.who.int/publications/i/item/who-ucn-ncd-20.1.

[4] National Institutes of Health What is Diabetes?. https://www.niddk.nih.gov/health-information/diabetes/overview/what-is-diabetes.

[5] (2020). National Diabetes Statistics Report. https://www.cdc.gov/diabetes/pdfs/data/statistics/national-diabetes-statistics-report.pdf.

[6] Association American Diabetes 2 (2021). Classification and Diagnosis of Diabetes: Standards of Medical Care in Diabetes-2021. Am. Diabetes Assoc..

[7] International Diabetes Federation (2013). Five questions on the IDF Diabetes Atlas. Diabetes Res. Clin. Pract..

[8] Tan S.Y., Mei Wong J.L., Sim Y.J., Wong S.S., Mohamed Elhassan S.A., Tan S.H., Ling Lim G.P., Rong Tay N.W., Annan N.C., Bhattamisra S.K. (2019). Type 1 and 2 diabetes mellitus: A review on current treatment approach and gene therapy as potential intervention. Diabetes Metab. Syndr. Clin. Res. Rev..

[9] Salehi B., Ata A., Kumar N.V.A., Sharopov F., Ramírez-Alarcón K., Ruiz-Ortega A., Ayatollahi S.A., Fokou P.V.T., Kobarfard F., Zakaria Z.A. (2019). Antidiabetic potential of medicinal plants and their active components. Biomolecules.

[10] Rubio-Almanza M., Cámara-Gómez R., Merino-Torres J.F. (2019). Obesity and type 2 diabetes: Also linked in therapeutic options. Endocrinol. Diabetes y Nutr..

[11] Serván P.R. (2018). Diet recomendations in diabetes and obesity. Nutr. Hosp..

[12] Xie F., Chan J.C.N., Ma R.C.W. (2018). Precision medicine in diabetes prevention, classification and management. J. Diabetes Investig..

[13] Rey D., Ospina L., Aragón D. (2015). Inhibitory effects of an extract of fruits of Physalis peruviana on some intestinal carbohydrases. Rev. Colomb. Cienc. Químico-Farm..

[14] Rey D., Miranda Sulis P., Alves Fernandes T., Gonçalves R., Silva Frederico M.J., Costa G.M., Aragon M., Ospina L.F., Mena Barreto Silva F.R. (2019). Astragalin augments basal calcium influx and insulin secretion in rat pancreatic islets. Cell Calcium.

[15] Monzón Daza G., Meneses Macías C., Forero A.M., Rodríguez J., Aragón M., Jiménez C., Ramos F.A., Castellanos L. (2021). Identification of α-Amylase and α-Glucosidase Inhibitors and Ligularoside A, a New Triterpenoid Saponin from Passiflora ligularis Juss (Sweet Granadilla) Leaves, by a Nuclear Magnetic Resonance-Based Metabolomic Study. J. Agric. Food Chem..

[16] Bernal C.A., Castellanos L., Aragón D.M., Martínez-Matamoros D., Jiménez C., Baena Y., Ramos F.A. (2018). Peruvioses A to F, sucrose esters from the exudate of Physalis peruviana fruit as α-amylase inhibitors. Carbohydr. Res..

[17] Durazzo A., Lucarini M., Santini A. (2021). Molecular Sciences Plants and Diabetes: Description, Role, Comprehension and Exploitation. Int. J. Mol. Sci..

[18] Ríos J.L., Francini F., Schinella G.R. (2015). Natural Products for the Treatment of Type 2 Diabetes Mellitus. Planta Med..

[19] Wang G.S., Hoyte C. (2019). Review of Biguanide (Metformin) Toxicity. J. Intensive Care Med..

[20] Liu X., Wei J., Tan F., Zhou S., Würthwein G., Rohdewald P. (2004). Antidiabetic effect of Pycnogenol^®^ French maritime pine bark extract in patients with diabetes type II. Life Sci..

[21] Vitetta L., Butcher B., Dal Forno S., Vitetta G., Nikov T., Hall S., Steels E. (2020). A Double-Blind Randomized Placebo-Controlled Study Assessing the Safety, Tolerability and Efficacy of a Herbal Medicine Containing Pycnogenol Combined with Papain and Aloe vera in the Prevention and Management of Pre-Diabetes. Medicines.

[22] Governa P., Baini G., Borgonetti V., Cettolin G., Giachetti D., Magnano A.R., Miraldi E., Biagi M. (2018). Phytotherapy in the management of diabetes: A review. Molecules.

[23] Artasensi A., Pedretti A., Vistoli G., Fumagalli L. (2020). Type 2 diabetes mellitus: A review of multi-target drugs. Molecules.

[24] AragónNovoa D. (2021). Passifloraligularis Juss. (Granadilla): Farmacológicos de una Estudios Químicos y Plantacon Potencial Terapéutico.

[25] Narayan T., Sanjay B., Bhaswati K., Simanta B., Sagar B., Barsha D., Yunus S., Seydur R., Aparajita G., Pratim D.P. (2020). A Herbal Composition from Premna herbacea, Useful for Prevention of Obesity and Type 2 Diabetes and a Method for Its Extraction. WO Patent.

[26] Saya O., Reiko T., Takuya Y., Norio I. (2020). Agent for Suppressing Carbohydrate Breakdown and Absorption. U.S. Patent.

[27] Lal H. (2020). A Novel Synergic Herbal Formulation for the Prevention and Treatment of Pre-Diabetes, Diabetes and Other Insulin Resistance Cases. WO Patent.

[28] Patanjali S., Sundaram C., Jayashree M., Anilkumar K., Sarala S., Bishwajit N., Ramesh V., Nitish N., Jayarajan K. (2019). Herbal Composition. SG Patent.

[29] Hexiao S., Guolong L. (2019). Composite Preparation for Improving Insulin Resistance, Preparation Method and Application. WO Patent.

[30] Vasant S.C., Anantrao S.V. (2019). Plant Fractions Having Anti-Pathogenesis Properties. WO Patent.

[31] Vieira K. (2019). Herbal Nutraceutical Formulation to Reduce Oxidative Stress, Viral and Microbial Infections, and Inflammation. U.S. Patent.

[32] Kasim T. (2019). The Use of Jerusalem Thorn Fruits as Herbal Tea For Diabetes Treatment. WO Patent.

[33] Diaz A.M.L., Uribe J.A.G., Torres N.T.Y., Palacio A.R.T., Lopez L.G.N. (2019). Agavaceae Extract Comprising Steroidal Saponins to Treat or Prevent Metabolic Disorder Related Pathologies. MX Patent.

[34] Eishin K., Shinya H. (2019). Gnetin c-Rich Melinjo Extract and Production Method Thereof. U.S. Patent.

[35] Housey G., Balash M.E. (2019). Plant Extracts with Anti-Diabetic and Other Useful Activities. AU Patent.

[36] Vijayabhanu S. (2019). Herbo-Mineral Formulation for Prevention, Treatment and Management of Diabetes and Method of Preparation Thereof. U.S. Patent.

[37] Lien P.-J., Sun C.-C., Kuo T.-C., Yang S.-C., Wu Y.-C., Chang F.-R., Hsieh T.-J., Tsai Y.-H., Du Y.-C. (2017). Extract of Toona Sinensis from Supercritical Fluid Extraction for Treating Diabetes and Metabolic Disease, the Preparation Method and the Use Thereof. U.S. Patent.

[38] Wah M.C., Zhen L., Xia Z., Xiaolei G. (2017). Compositions Comprising Cyclocarya Paliurus Extract and Preparation Method and Uses Thereof. U.S. Patent.

[39] Arvind S., Parikshit G., Aslam B., Somesh S. (2017). Herbal Composition for the Treatment of Metabolic Disorders. NZ Patent.

[40] Ko Y.-F., Jan M., Liau J.-C., Chang I.-T., Lee C.-S., Wang W.-C., Chiu W.-C., Chang C.Y., Lin C.-S., Wu T.-R. (2016). Method to Prepare Hirsutella Sinensis Polysaccharides Possessing Insulin-Sensitizing Properties and Applications Thereof. U.S. Patent.

[41] Horn G., Trimbo S. (2016). Compositions Capable of Enhancing Thermogenesis and Uses Thereof. U.S. Patent.

[42] Sik H.K., Yong C.J., Young P.S., Zhangjun H. (2016). Composition Comprising Suaeda Japonica for Preventing or Alleviating Diabetes. U.S. Patent.

[43] Mihaylivna M.S., Oleksandrivna S.A., Yurivna K.O. (2016). Herbal Species for Treatment of Diabetes Mellitus Type ll. UA Patent.

[44] Gaikwad S.B., Krishna Mohan G., Rani M.S. (2014). Phytochemicals for Diabetes Management. Pharm. Crop..

[45] Xia B., Shi X.C., Xie B.C., Zhu M.Q., Chen Y., Chu X.Y., Cai G.H., Liu M., Yang S.Z., Mitchell G.A. (2020). Urolithin A exerts antiobesity effects through enhancing adipose tissue thermogenesis in mice. PLoS Biol..

[46] Laddha A.P., Kulkarni Y.A. (2019). Tannins and vascular complications of Diabetes: An update. Phytomedicine.

[47] Wu S., Tian L. (2019). A new flavone glucoside together with known ellagitannins and flavones with anti-diabetic and anti-obesity activities from the flowers of pomegranate (Punica granatum). Nat. Prod. Res..

[48] Yuan T., Ferreira D., Seeram N. (2012). Punicatannins A and B: α-glucosidase inhibitory ellagitannins from pomegranate (Punica granatum) flowers. Planta Med..

[49] Lee M., Nam S.-H., Yoon H.-G., Kim S., You Y., Choi K.-C., Lee Y.-H., Lee J., Park J., Jun W. (2022). Fermented Curcuma longa L. Prevents Alcoholic Fatty Liver Disease in Mice by Regulating CYP2E1, SREBP-1c, and PPAR-α. J. Med. Food.

[50] Pujimulyani D., Yulianto W.A., Setyowati A., Arumwardana S., Kusuma H.S.W., Sholihah I.A., Rizal R., Widowati W., Maruf A. (2020). Hypoglycemic activity of curcuma mangga val. Extract via modulation of GLUT4 and ppar-γ mrna expression in 3T3-L1 adipocytes. J. Exp. Pharmacol..

[51] Mohammadi E., Behnam B., Mohammadinejad R., Guest P.C., Simental-Mendía L.E., Sahebkar A. (2021). Antidiabetic Properties of Curcumin: Insights on New Mechanisms. Advances in Experimental Medicine and Biology.

[52] Pivari F., Mingione A., Brasacchio C., Soldati L. (2019). Curcumin and type 2 diabetes mellitus: Prevention and treatment. Nutrients.

[53] Kotha R.R., Luthria D.L. (2019). Curcumin: Biological, pharmaceutical, nutraceutical, and analytical aspects. Molecules.

[54] Zeng L., Yu G., Hao W., Yang K., Chen H. (2021). The efficacy and safety of Curcuma longa extract and curcumin supplements on osteoarthritis: A systematic review and meta-analysis. Biosci. Rep..

[55] Jin T., Song Z., Weng J., Fantus I.G. (2018). Curcumin and other dietary polyphenols: Potential mechanisms of metabolic actions and therapy for diabetes and obesity. Am. J. Physiol.—Endocrinol. Metab..

[56] Scazzocchio B., Minghetti L., D’archivio M. (2020). Interaction between gut microbiota and curcumin: A new key of understanding for the health effects of curcumin. Nutrients.

[57] Nazaruk J., Borzym-Kluczyk M. (2015). The role of triterpenes in the management of diabetes mellitus and its complications. Phytochem. Rev..

[58] Zhong Y.Y., Chen H.S., Wu P.P., Zhang B.J., Yang Y., Zhu Q.Y., Zhang C.G., Zhao S.Q. (2019). Synthesis and biological evaluation of novel oleanolic acid analogues as potential α-glucosidase inhibitors. Eur. J. Med. Chem..

[59] Ding H., Hu X., Xu X., Zhang G., Gong D. (2018). Inhibitory mechanism of two allosteric inhibitors, oleanolic acid and ursolic acid on α-glucosidase. Int. J. Biol. Macromol..

[60] Chen W., Balan P., Popovich D.G. (2019). Review of ginseng anti-diabetic studies. Molecules.

[61] Sun Y., Gao H.Y., Fan Z.Y., He Y., Yan Y.X. (2020). Metabolomics signatures in type 2 diabetes: A systematic review and integrative analysis. J. Clin. Endocrinol. Metab..

[62] Kim Y.A., Keogh J.B., Clifton P.M. (2018). Probiotics, prebiotics, synbiotics and insulin sensitivity. Nutr. Res. Rev..

[63] Judge A., Dodd M.S. (2020). Metabolism. Essays Biochem..

[64] Tokarz V.L., MacDonald P.E., Klip A. (2018). The cell biology of systemic insulin function. J. Cell Biol..

[65] Sims E.K., Carr A.L.J., Oram R.A., DiMeglio L.A., Evans-Molina C. (2021). 100 years of insulin: Celebrating the past, present and future of diabetes therapy. Nat. Med..

[66] Devangan S., Varghese B., Johny E., Gurram S., Adela R. (2021). The effect of Gymnema sylvestre supplementation on glycemic control in type 2 diabetes patients: A systematic review and meta-analysis. Phyther. Res..

[67] Sandech N., Jangchart R., Komolkriengkrai M., Boonyoung P., Khimmaktong W. (2021). Efficiency of Gymnema sylvestre-derived gymnemic acid on the restoration and improvement of brain vascular characteristics in diabetic rats. Exp. Ther. Med..

[68] Al-Romaiyan A., Liu B., Persaud S., Jones P. (2020). A novel Gymnema sylvestre extract protects pancreatic beta-cells from cytokine-induced apoptosis. Phyther. Res..

[69] Abdulrahman M.D., Zakariya A.M., Hama H.A., Hamad S.W., Al-Rawi S.S., Bradosty S.W., Ibrahim A.H. (2022). Ethnopharmacology, Biological Evaluation, and Chemical Composition of Ziziphus spina- christi (L.) Desf.: A Review. Adv Pharmacol. Pharm. Sci..

[70] Kane J.P., Pullinger C.R., Goldfine I.D., Malloy M.J. (2021). Dyslipidemia and diabetes mellitus: Role of lipoprotein species and interrelated pathways of lipid metabolism in diabetes mellitus. Curr. Opin. Pharmacol..

[71] Athyros V.G., Stavropoulos K., Georgianou E., Katsimardou A., Karagiannis A., Michael Doumas Konstantinos P Imprialos (2018). Diabetes and lipid metabolism. Hormones.

[72] Manukumar H.M., Shiva Kumar J., Chandrasekhar B., Raghava S., Umesha S. (2017). Evidences for diabetes and insulin mimetic activity of medicinal plants: Present status and future prospects. Crit. Rev. Food Sci. Nutr..

[73] Choi H.M., Doss H.M., Kim K.S. (2020). Multifaceted physiological roles of adiponectin in inflammation and diseases. Int. J. Mol. Sci..

[74] Fang H., Judd R.L. (2018). Adiponectin regulation and function. Compr. Physiol..

[75] Unuofin J.O., Lebelo S.L. (2020). Antioxidant Effects and Mechanisms of Medicinal Plants and Their Bioactive Compounds for the Prevention and Treatment of Type 2 Diabetes: An Updated Review. Oxid. Med. Cell. Longev..

[76] EMA Assessment Report on Panax Ginseng C.A. https://www.ema.europa.eu/en/documents/herbal-report/final-assessment-report-panax-ginseng-ca-meyer-radix_en.pdf.

[77] Muñoz-Garach A., Diaz-Perdigones C., Tinahones F.J. (2016). Gut microbiota and type 2 diabetes mellitus. Endocrinol. Nutr..

[78] Hills R.D., Pontefract B.A., Mishcon H.R., Black C.A., Sutton S.C., Theberge C.R. (2019). Gut microbiome: Profound implications for diet and disease. Nutrients.

[79] Magne F., Gotteland M., Gauthier L., Zazueta A., Pesoa S., Navarrete P., Balamurugan R. (2020). The firmicutes/bacteroidetes ratio: A relevant marker of gut dysbiosis in obese patients?. Nutrients.

[80] Betz M.J., Enerbäck S. (2018). Targeting thermogenesis in brown fat and muscle to treat obesity and metabolic disease. Nat. Rev. Endocrinol..

[81] Pfeiffer A.F.H., Klein H.H. (2014). Therapie des diabetes mellitus typ 2. Dtsch. Arztebl. Int..

[82] Abrilla A.A., Pajes A.N.N.I., Jimeno C.A. (2021). Metformin extended-release versus metformin immediate-release for adults with type 2 diabetes mellitus: A systematic review and meta-analysis of randomized controlled trials. Diabetes Res. Clin. Pract..

[83] Li H., Qi J., Li L. (2019). Phytochemicals as potential candidates to combat obesity via adipose non-shivering thermogenesis. Pharmacol. Res..

[84] Chukwuma C.I., Matsabisa M.G., Ibrahim M.A., Erukainure O.L., Chabalala M.H., Islam M.S. (2019). Medicinal plants with concomitant anti-diabetic and anti-hypertensive effects as potential sources of dual acting therapies against diabetes and hypertension: A review. J. Ethnopharmacol..

[85] Anderson L.A. (2022). Drug Names and Their Pharmaceutical Salts—Clearing Up the Confusion. https://www.drugs.com/article/pharmaceutical-salts.html.

[86] de Freitas C.D.T., Nishi B.C., do Nascimento C.T.M., Silva M.Z.R., Bezerra E.H.S., Rocha B.A.M., Grangeiro T.B., de Oliveira J.P.B., Souza P.F.N., Ramos M.V. (2020). Characterization of Three Osmotin-Like Proteins from Plumeria rubra and Prospection for Adiponectin Peptidomimetics. Protein Pept. Lett..

[87] Sushma M., Raju Y., Sundaresan C.R., Vandana K.R., Kumar N., Chowdary V. (2014). Transmucosal Delivery of Metformin- A Comprehensive Study. Curr. Drug Deliv..

[88] Modgill V., Garg T., Goyal A., Rath G. (2015). Transmucosal Delivery of Linagliptin for the Treatment of Type- 2 Diabetes Mellitus by Ultra-Thin Nanofibers. Curr. Drug Deliv..

[89] Huang F.J., Lan K.C., Kang H.Y., Liu Y.C., Der Hsuuw Y., Chan W.H., Huang K.E. (2013). Effect of curcumin on in vitro early post-implantation stages of mouse embryo development. Eur. J. Obstet. Gynecol. Reprod. Biol..

[90] Felter S.P., Vassallo J.D., Carlton B.D., Daston G.P. (2006). A safety assessment of coumarin taking into account species-specificity of toxicokinetics. Food Chem. Toxicol..

[91] Miura T., Uehara S., Shimizu M., Murayama N., Suemizu H., Yamazaki H. (2021). Roles of human cytochrome P450 1A2 in coumarin 3,4-epoxidation mediated by untreated hepatocytes and by those metabolically inactivated with furafylline in previously transplanted chimeric mice. J. Toxicol. Sci..

[92] Hsieh C.Y.J., Sun M., Osborne G., Ricker K., Tsai F.C., Li K., Tomar R., Phuong J., Schmitz R., Sandy M.S. (2019). Cancer Hazard Identification Integrating Human Variability: The Case of Coumarin. Int. J. Toxicol..

[93] Zjablovskaja P., Danek P., Kardosova M., Alberich-Jorda M. (2018). Proliferation and differentiation of murine myeloid precursor 32d/g-csf-r cells. J. Vis. Exp..

[94] Pandey S., Dvorakova M.C. (2019). Future Perspective of Diabetic Animal Models. Endocr. Metab. Immune Disord.—Drug Targets.

[95] Kottaisamy C.P.D., Raj D.S., Prasanth Kumar V., Sankaran U. (2021). Experimental animal models for diabetes and its related complications—A review. Lab. Anim. Res..

[96] Lenzen S. (2008). The mechanisms of alloxan- and streptozotocin-induced diabetes. Diabetologia.

[97] NIH Search of: Herbal. Diabetes Mellitus. Adult—List Results—ClinicalTrials.gov 2022. https://www.clinicaltrials.gov/ct2/results?term=herbal&cond=Diabetes+Mellitus&age=1#.

[98] Gammone M.A., D’Orazio N. (2015). Anti-obesity activity of the marine carotenoid fucoxanthin. Mar. Drugs.

[99] Bannu S.M., Lomada D., Gulla S., Chandrasekhar T., Reddanna P., Reddy M.C. (2019). Potential Therapeutic Applications of C-Phycocyanin. Curr. Drug Metab..

[100] Seo Y.J., Kim K.J., Choi J., Koh E.J., Lee B.Y. (2018). Spirulina maxima extract reduces obesity through suppression of adipogenesis and activation of browning in 3T3-L1 cells and high-fat diet-induced obese mice. Nutrients.

[101] Wu T.R., Lin C.S., Chang C.J., Lin T.L., Martel J., Ko Y.F., Ojcius D.M., Lu C.C., Young J.D., Lai H.C. (2019). Gut commensal Parabacteroides goldsteinii plays a predominant role in the anti-obesity effects of polysaccharides isolated from Hirsutella sinensis. Gut.

[102] Lu Z., Li S., Sun R., Jia X., Xu C., Aa J., Wang G. (2019). Hirsutella sinensis Treatment Shows Protective Effects on Renal Injury and Metabolic Modulation in db/db Mice. Evid.-Based Complement. Altern. Med..

[103] Jafari M., Forootanfar H., Ameri A., Foroutanfar A., Adeli-Sardou M., Rahimi H.R., Najafi A., Zangiabadi N., Shakibaie M. (2019). Antioxidant, cytotoxic and hyperalgesia-suppressing activity of a native Shilajit obtained from Bahr Aseman mountains. Pak. J. Pharm. Sci..

[104] Pathiraja P.M.Y.S., Ranatunga Y.M.M.K., Herapathdeniya S.K.M.K., Gunawardena S.H.P. (2020). Swarna Makshika Bhasma preparation using an alternative heating method to traditional Varaha Puta. J. Ayurveda Integr. Med..

[105] Kantak S., Rajurkar N., Adhyapak P. (2020). Synthesis and characterization of Abhraka (mica) bhasma by two different methods. J. Ayurveda Integr. Med..

[106] Singh A., Ota S., Srikanth N., Galib R., Bojja S., Dhiman K.S. (2018). Application of Spectroscopic and Chromatographic Methods for Chemical Characterization of an Ayurvedic Herbo-Mineral Preparation: Maha Yograja Guggulu. J. Evidence-Based Integr. Med..

[107] Pareek A., Bhatnagar N. (2020). Physico-chemical characterization of traditionally prepared Yashada bhasma. J. Ayurveda Integr. Med..

